# Congenital aerodigestive fistula with extraordinarily delayed adult presentation: a case report and review of endoscopic closure for bronchoesophageal fistula

**DOI:** 10.3389/fmed.2026.1887528

**Published:** 2026-07-02

**Authors:** Ning Liang, Jiang-Long Liu, Ke Zhan, Yi-Shi Li, Yang Bai

**Affiliations:** 1 Department of Anesthesiology, The First Affiliated Hospital of Chongqing Medical University, Chongqing, China; 2 Department of Respiratory and Critical Care Medicine, The People's Hospital of Neijiang Dongxing District, Neijiang, Sichuan, China; 3 Department of Gastroenterology, The First Affiliated Hospital of Chongqing Medical University, Chongqing, China; 4 Department of Respiratory and Critical Care Medicine, The First Affiliated Hospital of Chongqing Medical University, Chongqing, China

**Keywords:** aerodigestive fistula, argon plasma coagulation, case report, congenital bronchoesophageal fistula, endoscopic closure, over-the-scope clip

## Abstract

**Background:**

Congenital aerodigestive fistulae (ADF) represent extremely rare developmental malformations derived from disrupted embryological separation of the primitive foregut, which can be classified by lesion location into tracheoesophageal fistulae, bronchoesophageal fistulae (BEF), and other subtypes. While typically diagnosed in neonates, isolated BEF without esophageal atresia (“H-type”) may remain undetected for decades, presenting in adulthood with chronic, stereotyped symptoms. Endoscopic interventions for congenital BEF remain poorly characterized, with limited evidence regarding patient selection and technique optimization.

**Case presentation:**

A 58-year-old man presented with a 40-year history of persistent, swallowing-induced cough, accompanied by expectoration of white mucoid sputum. Initial chest computed tomography at an outside hospital identified a left lower lobe mass suspicious of lung cancer complicated by obstructive pneumonia. Bronchoscopy confirmed an esophageal–left main bronchial fistula, and endobronchial ultrasound-guided biopsy demonstrated acute and chronic inflammation with fibrous hyperplasia. Repeat imaging at our institution demonstrated a direct, upward fistulous tract between the mid-esophagus and left main bronchus, consistent with Braimbridge and Keith Type II congenital BEF. A multidisciplinary consensus deemed surgical repair high-risk with uncertain efficacy, and the patient declined operative intervention. Combined bronchoscopy and esophagoscopy under general anesthesia confirmed a 2-mm fistula orifice 2 cm below the left main carina. The fistula was successfully closed using argon plasma coagulation-mediated de-epithelialization followed by over-the-scope clip (OTSC) deployment, with immediate resolution of swallowing-induced cough. At the 10-month follow-up, esophagography showed no contrast extravasation, computed tomography confirmed no persistent fistulous communication, and esophagoscopy demonstrated granulation tissue with white scarring and no identifiable fistula orifice.

**Conclusion:**

This case illustrates the potential for extraordinarily delayed presentation of congenital BEF in adulthood and highlights the importance of maintaining a high index of suspicion for this rare anomaly in patients with chronic, liquid-induced paroxysmal cough. Combined endoscopic de-epithelialization and OTSC closure appears to be an effective, minimally invasive alternative to surgical repair in eligible patients with congenital BEF. More clinical studies are warranted to develop consensus-based guidelines for the management of benign ADF.

## Introduction

Aerodigestive fistulae (ADF) represent abnormal communications between the respiratory and gastrointestinal tracts, including tracheoesophageal fistulae, bronchoesophageal fistulae (BEF), and other rare anatomical variants (1). Congenital ADF derive from dysregulated embryological separation of the primitive foregut (2, 3) with an estimated incidence of one case in 3,000 to 4,000 live births (4). However, isolated congenital BEF without esophageal atresia, the H-type malformation, is an evidently rare subset, which frequently escapes early recognition (5). The clinical manifestations of congenital BEF are characterized by considerable heterogeneity. Neonatal cases typically present with feeding intolerance, recurrent aspiration pneumonia, or respiratory distress, facilitating early diagnosis (6). Conversely, isolated congenital BEF in adults often manifests with chronic liquid-induced paroxysmal cough or recurrent aspiration pneumonia. The delayed onset of symptoms may result from an upward fistula course, a complete membranous occlusion, or temporary functional closure of the fistula during swallowing (5). This physiological adaptation may delay symptom onset for decades, with reported intervals between symptom onset and definitive diagnosis ranging from 6 months to 50 years (5, 7).

Surgical resection with primary fistula repair remains the standard treatment for congenital ADF, yielding durable closure in over 90% of appropriately selected patients (8–10). However, it carries substantial perioperative risks, particularly in those with severe adhesions, malnutrition, or serious cardiopulmonary comorbidities. Moreover, some patients refuse surgery or have anatomical variations that impede surgical exposure. Endoscopic interventions have emerged as minimally invasive alternatives, including temporary or permanent stenting, mucosal de-epithelialization, tissue sealants, and mechanical closure devices (11). Notably, the over-the-scope clip (OTSC) system, constructed from super-plastic nitinol in a “bear-claw” design, provides full-thickness tissue compression that surpasses conventional through-the-scope clips, enabling airtight closure of gastrointestinal lesions up to 10 mm in diameter (12). Nevertheless, data endorsing endoscopic interventions for congenital ADF remains confined to isolated case reports and small retrospective series, demonstrating variable success rates and inadequately defined patient selection criteria (11, 13). Herein, we report a case of Braimbridge and Keith Type II congenital BEF diagnosed in a 58-year-old man after 40 years of liquid-induced paroxysmal cough, successfully managed with combined argon plasma coagulation (APC)–mediated de-epithelialization and OTSC closure via the transesophageal approach. This case illustrates: (i) the potential for extraordinarily delayed presentation of congenital BEF in adulthood; (ii) the diagnostic utility of combined bronchoscopy and esophagoscopy with methylene blue confirmation; and (iii) a multidisciplinary, minimally invasive endoscopic strategy as a feasible alternative to surgical repair in carefully selected patients.

## Case presentation

A 58-year-old man presented with a 40-year history of paroxysmal cough immediately triggered by drinking liquids, accompanied by expectoration of white mucoid sputum. He denied having chills, fever, chest tightness, chest pain, or hemoptysis throughout this period. Three months prior to the index admission, his symptoms worsened in both frequency and severity, prompting him to seek definitive diagnosis and treatment. At an outside hospital, chest computed tomography (CT) identified a small parenchymal mass in the dorsal segment and anteromedial basal segments of the left lower lobe, with associated perilesional consolidation highly suggestive of primary lung cancer with obstructive pneumonia. Subsequent bronchoscopy confirmed an esophageal–left main bronchial fistula with inflammatory changes in the left bronchial tree. Endobronchial ultrasound-guided transbronchial lung biopsy in the medial basal segment demonstrated acute and chronic inflammation with fibrous hyperplasia. The patient received moxifloxacin for anti-infective treatment but refused nasogastric tube insertion. A tentative diagnosis of BEF involving the left main bronchus was established, and the patient was transferred to our institution for further treatment.

**Figure 1:**
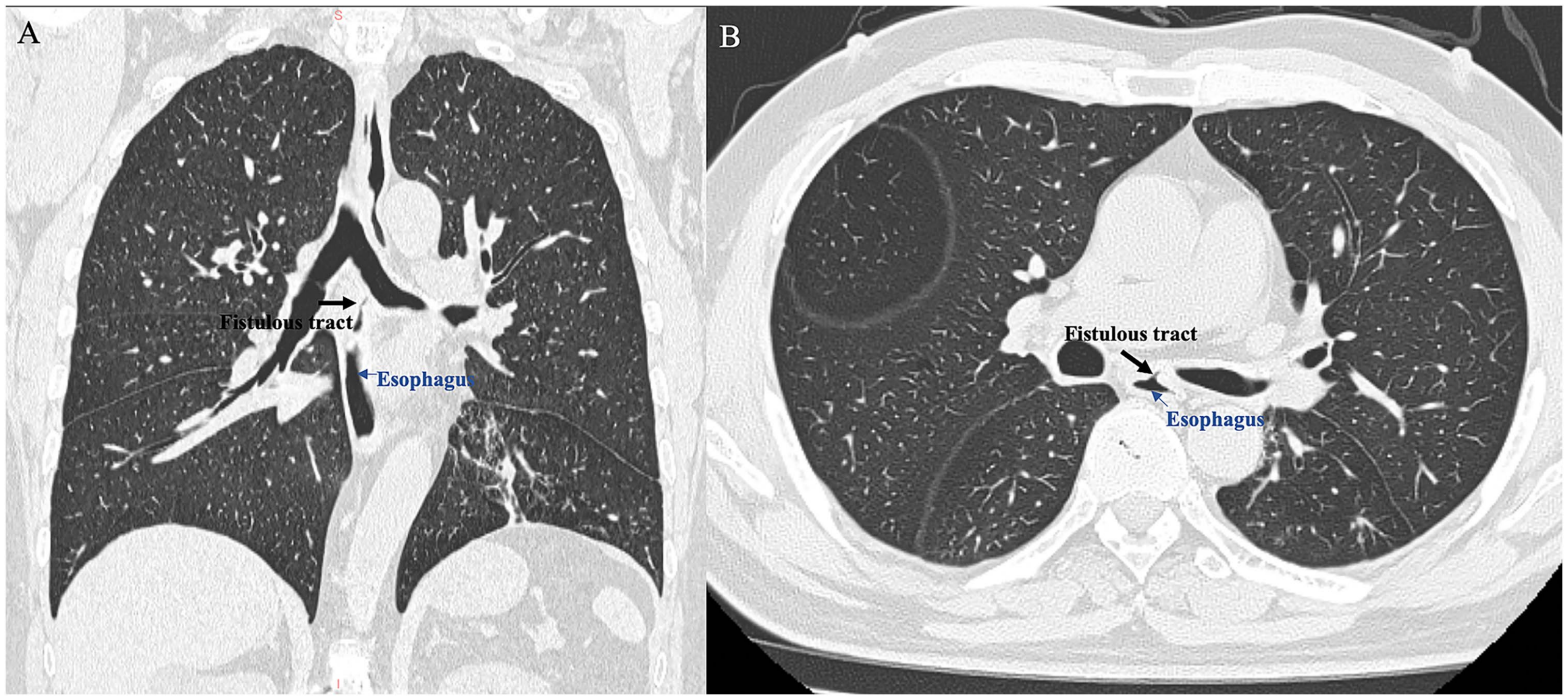
Preoperative thoracic imaging. **(A)** Coronal and **(B)** axial chest computed tomography demonstrating a direct, upward fistulous tract (black arrow) between the mid-esophagus (blue arrow) and the left main bronchus, consistent with the Braimbridge and Keith Type II congenital bronchoesophageal fistula.

The patient had a 40-pack-year smoking history (about six cigarettes per day) but had quit before admission. He also reported 40 years of regular alcohol usage (mostly white liquor at 150 mL daily), which he had ceased prior to admission. Past surgical history included appendectomy and lumbar spine surgery following traumatic injury. There was no known family history of congenital malformations or malignancy. Psychosocial history was unremarkable except for long-term dietary restriction due to fear of coughing after drinking liquids. Over four decades, he had not received any specific intervention for his swallowing-related cough. Symptomatic treatment was occasionally sought at local clinics, but no formal diagnostic workup had been performed. The patient’s temperature was 36.6 °C, heart rate of 82 beats per minute, respiratory rate of 21 breaths per minute, and blood pressure of 132/76 mmHg on admission. Auscultation revealed diminished bilateral breath sounds with no wheezes, rales or rhonchi. Laboratory tests, including the autoimmune disease screening panel, were all within normal limits. Repeat chest CT with multiplanar reconstruction indicated a direct, upward fistulous tract between the mid-esophagus and the left main bronchus (Figure 1), consistent with Braimbridge and Keith Type II congenital BEF. Notably, the left lower lobe parenchymal lesion exhibited significant improvement after antibiotic therapy and fasting, indicating a diagnosis of secondary aspiration pneumonia.

**Figure 2:**
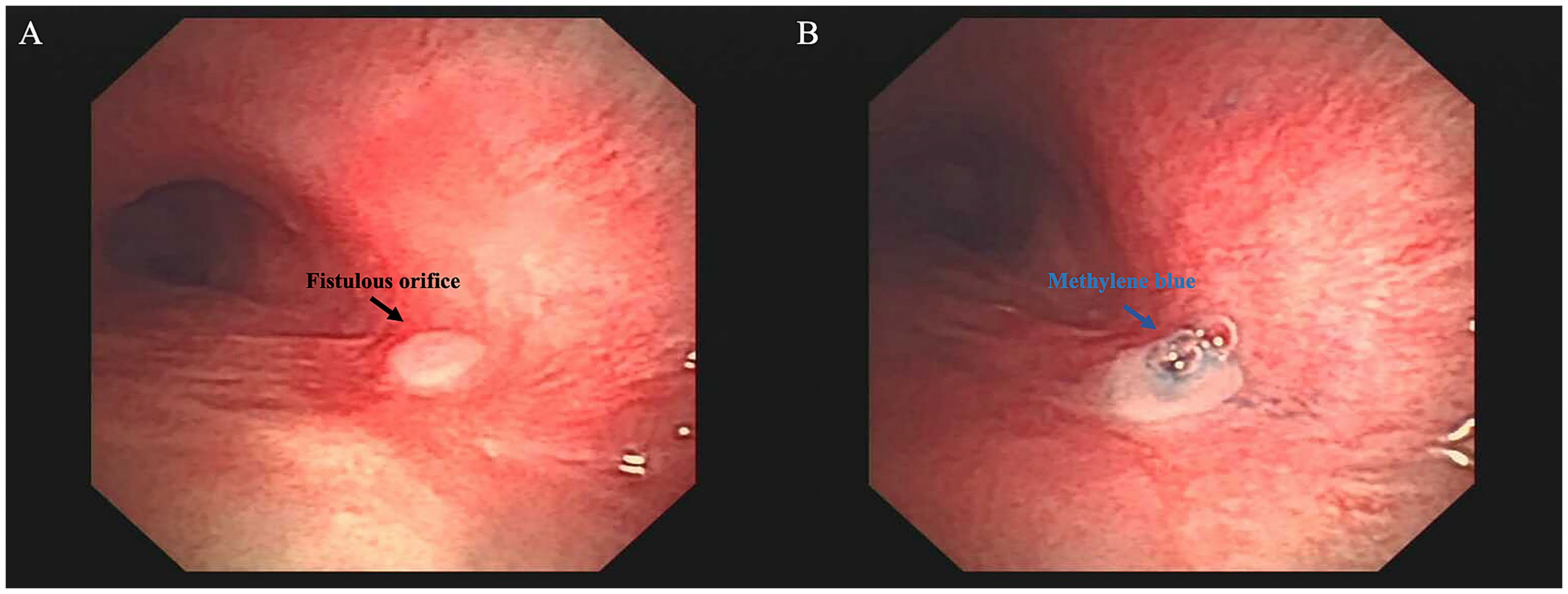
Preoperative bronchoscopy. **(A)** Bronchoscopic view identifying a small fistula orifice measuring 2 mm in diameter, located 2 cm below the left main carina and covered by adjacent white necrotic material (black arrow). **(B)** Methylene blue instilled into the esophagus extravasated and bubbled at the fistula orifice (blue arrow), confirming communication.

Bronchoscopy found a small fistula orifice (2 mm in diameter) covered with adjacent white necrotic tissue, 2 cm distal to the left main carina (Figure 2A). Communication was confirmed by extravasation of methylene blue (1%) instilled into the esophagus and formation of bubbles at the fistula orifice (Figure 2B). A multidisciplinary consensus meeting was conducted among specialists in pulmonology, thoracic surgery, gastroenterology, radiology, and anesthesiology. Critical considerations were: (1) 40-year clinical course of benignity suggesting a congenital etiology; (2) histopathological confirmation of non-malignancy; (3) high operative risk and patient preference; and (4) small fistula favoring endoscopic closure. Surgical treatment was considered high risk and of uncertain efficacy, and the patient declined the surgery. The multidisciplinary consensus recommended simultaneous esophagoscopy and bronchoscopy under general anesthesia via the laryngeal mask airway for thorough evaluation and endoscopic fistula closure.

**Figure 3:**
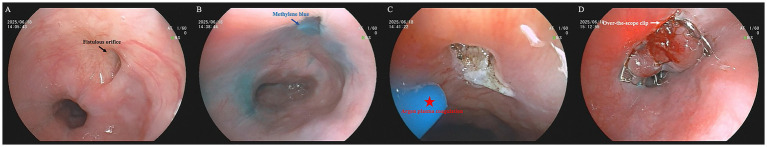
Endoscopic closure of congenital bronchoesophageal fistula using argon plasma coagulation and the over-the-scope clip. **(A)** Esophagoscopy revealing a focal depression with smooth mucosa in the mid-esophagus (28 cm from the incisors), corresponding to the fistula orifice (black arrow). **(B)** Methylene blue sprayed at the bronchial side of the fistula extravasated into the esophageal depression (blue arrow), confirming communication. **(C)** The perilesional mucosa around the fistula orifice was ablated with argon plasma coagulation (red asterisk) to promote healing. **(D)** The fistula was successfully closed using an over-the-scope clip (white arrow), confirmed by the repeat methylene blue injection at the bronchial side with no further dye extravasation from the esophageal side.

**Figure 4:**
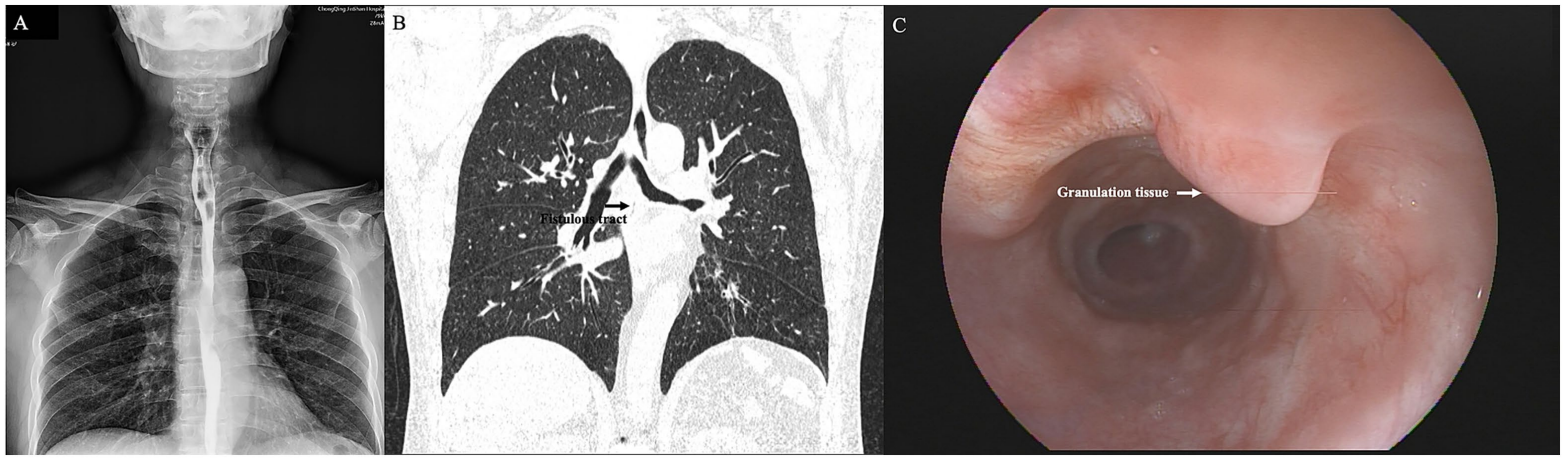
Postoperative follow-up at 10 months. **(A)** Esophagography showing no contrast extravasation. **(B)** Coronal thoracic computed tomography demonstrating no persistent communication between the esophagus and left main bronchus, with marked reduction in the residual fistulous tract (black arrow) compared with prior imaging. **(C)** Esophagoscopy revealing granulation tissue (white arrow) at the previous fistula site (28 cm from the incisors), surrounded by white scarring, with no identifiable fistula orifice on repeated inspection.

During esophagoscopy, a focal mucosal depression was identified at 28 cm from the incisors in the mid-esophagus, corresponding to the fistula orifice (Figure 3A). Methylene blue injected via the bronchial side extravasated into this esophageal lesion, demonstrating communication (Figure 3B). Perilesional mucosa within a 5-mm radius was ablated with APC (ERBE Elektromedizin GmbH, Germany; APC 2 module, forced coagulation mode, power 40 W power, argon gas flow rate 1.5 L/min) to strip off the epithelial lining and stimulate granulation tissue development (Figure 3C). Application was performed using intermittent, non-contact pulses (1–2 s per pulse) with circumferential ablation of the perilesional mucosa. Thereafter, the fistula was closed transesophageally with an OTSC (Ningbo SensCure Biotechnology, China; 10 mm diameter, 6 mm cap depth, blunt tooth profile) under general anesthesia (Figure 3D). The fistula orifice and surrounding de-epithelialized mucosa were suctioned into the transparent cap mounted on the endoscope tip, and the clip was deployed via the hand wheel mechanism once adequate tissue approximation was confirmed endoscopically, achieving full-thickness tissue compression. Successful closure was confirmed by repeat methylene blue injection via the bronchial side, with no dye extravasation observed from the esophageal side. Postoperatively, the patient was kept nil per os with acid suppression and nutritional support for 48 h. Enteral nutrition was resumed after the passage of flatus, provided there was no abdominal distension, nausea, vomiting, or other adverse reactions. The patient’s swallowing-induced cough resolved completely immediately after the endoscopic closure, without procedure-related complications, recurrent respiratory infections, or dysphagia. He tolerated oral liquids and foods without any aspiration symptoms. This intervention substantially improved his quality of life. Esophagography at 10 months post-treatment showed no contrast extravasation (Figure 4A). Coronal chest CT confirmed the absence of persistent bronchoesophageal communication and a marked reduction in the residual fistulous tract (Figure 4B) compared with prior imaging. Esophagoscopy showed granulation tissue at the previous fistula location (28 cm from the incisors), surrounded by white scarring, with no identifiable fistula orifice on repeated inspection (Figure 4C).

## Discussion

ADF can be broadly classified as acquired or congenital. Acquired fistulas typically arise from malignant infiltration, thoracic trauma, inflammatory disorders or iatrogenic injury (14–17). We systematically excluded the possible acquired causes of ADF in this case. Pulmonary malignancy was ruled out based on negative pathological results of biopsy specimens and significant resolution of pulmonary inflammatory lesions after standardized anti-infective treatment. Chronic persistent pulmonary infection and autoimmune inflammatory diseases were excluded by normal serological laboratory indicators, negative autoimmune antibody screening results, and the absence of corresponding typical clinical manifestations. In addition, the patient had no definite history of thoracic trauma, esophageal or airway surgery, or thoracic radiotherapy, which completely excluded traumatic and iatrogenic fistula formation factors. Combined with the patient’s long-term atypical chronic symptoms since adolescence, the final diagnosis of congenital BEF was confirmed. Congenital ADF represent rare developmental anomalies (6), but constitute the most common benign, non-iatrogenic subtype (7). This patient was diagnosed with congenital BEF based on multiple convincing clinical characteristics. First, the 40-year history of liquid-induced paroxysmal cough is an extraordinarily prolonged clinical course inconsistent with acute acquired fistula formation. Secondly, a thorough evaluation indicated no predisposing factors for acquired ADF, such as thoracic injury, recent esophageal or airway surgery, mediastinal radiation, or severe necrotizing pulmonary infection. Third, histological examination showed only chronic inflammation with fibrous hyperplasia, excluding a malignant origin. Finally, bronchoscopic and radiological findings confirmed the existence of a short, direct fistulous tract between the mid-esophagus and left main bronchus, anatomically consistent with Braimbridge and Keith Type II congenital BEF, the most frequent subtype (18).

The delayed presentation in this adult patient warrants specific consideration. The upward orientation of the fistulous tract from the middle esophagus toward the left main bronchus likely allowed a prolonged asymptomatic or mildly symptomatic course. This anatomical structure may enable a temporary functional closure during esophageal peristalsis and swallowing, thereby decreasing aspiration severity and delaying symptom onset (5). Published literature reports symptom delay ranging from 6 months to 50 years prior to definitive diagnosis for congenital BEF (5, 7); this case exemplifies this phenomenon. Initial imaging findings of pulmonary parenchymal lesions were attributed to secondary aspiration pneumonia from chronic fistulous communication, rather than fistula formation induced by primary lung infection (19).

In this case, the definitive diagnosis of BEF was confirmed by direct visualization of the fistula orifice during combined esophagoscopy and bronchoscopy, with methylene blue injection demonstrating communication. In pediatric patients, flexible bronchoscopy with methylene blue injection appears superior to conventional radiological examinations including esophagography and contrast radiography (20, 21). This is largely attributed to poor patient compliance, small fistulous tracts, and unsatisfactory contrast opacification commonly seen in children. In contrast, radiological examinations remain the most widely adopted diagnostic method for benign, non-iatrogenic BEF, in adult populations, with a reported diagnostic sensitivity approaching 97.4% (7). The present case highlights the complementary value of cross-sectional imaging, contrast studies, and endoscopic examination in establishing an accurate diagnosis for congenital ADF.

Congenital ADF are embryological anomalies deriving from dysregulated foregut development (2). A tracheal band appears on the ventral surface of the primitive foregut at around the 3-mm embryonic stage. Rapid caudal elongation and separation of the trachea from the esophagus generally occur by the 5-mm stage. Discoordination between these temporally coordinated developmental processes causes chronic airway-esophageal adhesion and eventually fistula formation. The exact anatomical location of congenital ADF is determined by the extent of tracheoesophageal separation prior to caudal elongation of the trachea (3). Developmental biology has elucidated the molecular mechanisms underlying this congenital malformation. The dorsoventral patterning of the anterior foregut endoderm is modulated by mutually antagonistic transcription factors, with SOX2 marking the dorsal esophageal lineage and NKX2-1 specifying the ventral respiratory lineage (22). Identification of such genetic aberrations may facilitate early prenatal or neonatal diagnosis, thereby enabling prompt intervention before the onset of recurrent aspiration pneumonia and nutritional impairment (23).

Management strategies for congenital ADF remain individualized depending on symptom severity, fistula size, anatomical location, and comorbidity profile (7). Historically, surgical resection with primary fistula repair has served as the gold standard for symptomatic benign ADF, achieving a long-term cure rate of over 90% while carrying significant perioperative risks, particularly in patients with extensive inflammatory adhesions, inadequate nutritional status, or significant cardiopulmonary comorbidities (8–10). These potential perioperative risks include iatrogenic central airway compromise when fistulas are located within 3 cm of the carina, unpredictable hemorrhage from the dense peribronchial vascular plexus, and traction injury to the vagus or recurrent laryngeal nerves during mediastinal dissection; chronic inflammation associated with long-standing fistulas can also lead to severe mediastinal fibrosis and adhesions that increase the risk of visceral injury and postoperative fistula recurrence (24). In the present case, multiple factors collectively rendered conventional surgical repair high-risk. First, the fistula orifice was found only 2 cm below the tracheal carina at the junction of mid-esophagus and left main bronchus, an anatomically unfavorable location where thoracotomy-based dissection could potentially cause stenosis of the left main bronchial lumen. Second, 40 years of persistent fistulous irritation triggered local fibrous hyperplasia, resulting in dense perilesional fibrosis and mediastinal adhesion that complicated surgical separation. In addition, the patient’s decades-long heavy tobacco and alcohol consumption impaired baseline pulmonary reserve and reduced tolerance to single-lung ventilation required for thoracic surgery. The patient declined surgical repair, prompting endoscopic closure as the initial therapeutic strategy.

Endoscopic interventions have emerged as effective minimally invasive therapeutic options for benign ADF, including temporary stent placement, mucosal de-epithelialization, tissue sealant injection, and combined de-epithelialization with sealant injection (11). Endoscopic stenting for benign ADF has only been documented in sporadic case reports and small case series (16, 25–27). For fistulae complicated by active inflammation, stenting serves as a mechanical scaffold to promote granulation tissue ingrowth and reduce air leakage, thereby facilitating fistula closure (16, 25). Nevertheless, endoscopic stenting has shown limited therapeutic efficacy in managing congenital ADF, with unfavorable clinical outcomes and complications (11). Mucosal de-epithelialization serves as an essential preparatory step before endoscopic closure of congenital ADF. This procedure refers to mechanical or chemical ablation of the epithelial lining around the fistulous tract, transforming chronic, epithelialized tracts into fresh, healing wounds. By facilitating granulation tissue proliferation and fibrous tissue hyperplasia, this maneuver markedly improves the success rate of subsequent closure modalities, such as endoscopic clips, tissue sealants and suturing. Multiple endoscopic modalities can achieve effective de-epithelialization. The most widely employed technique, APC, ablates the fistula orifice and adjacent mucosa (generally within a 5–10 mm radius) at power settings of 40–60 W, resulting in a uniform cauterized defect that promotes tissue adhesion when the fistula is subsequently closed with clips, flaps, or sealants (28). Mechanical abrasion via an endoscopic brush or wire serves as an alternative or supplementary method, sometimes combined with APC or sealants to induce deeper tissue injury and enhance healing (29). Chemical de-epithelialization with agents such as trichloroacetic acid is reserved for pediatric or delicate cases where thermal ablation is contraindicated (13). A recent meta-analysis of endoscopic closure for benign ADF reported that de-epithelialization (via APC, laser, brush, or trichloroacetic acid) was performed in more than 70% of cases prior to definitive closure, with the combination of de-epithelialization combined with sealant application yielding a high clinical success rate of 77.4% (11).

Endoscopic interventions can be performed via either the transbronchial or transesophageal route, with the latter generally preferred owing to fundamental anatomical superiority. The esophageal wall is markedly thicker than the tracheobronchial wall, permitting a broader spectrum of therapeutic maneuvers, such as mucosal ablation, clip deployment, and sealant injection. These strategies enable optimal tissue approximation and more sustained fistula closure (30). Moreover, transesophageal mechanical closure devices such as OTSC can be spontaneously expelled via the gastrointestinal tract, obviating the need for repeat endoscopic retrieval (12). Conversely, endobronchial implants carry high risks of migration, granulation hyperplasia, and mucosal infection, which frequently necessitate repeated bronchoscopic interventions (1, 16).

Originally designed to treat gastrointestinal leaks, perforations, fistulae, and refractory bleeding, the OTSC is a highly effective minimally invasive endoscopic device (31). The unique design and functional features contribute to its superior performance. Fabricated from super-elastic nitinol alloy and designed in a sturdy bear-claw shape, the OTSC enables full-thickness tissue compression, which is markedly superior to that achieved by conventional through-the-scope clips, thereby achieving airtight, leak-proof closure (32). Moreover, its variable clip sizes and suction-assisted cap design allow sufficient tissue capture, enabling uniform force distribution across the defect and reducing the risk of clip dislodgement. Multiple tooth profiles such as blunt, spiked, and *the success rate* elongated also make it adaptable to diverse tissue textures and fistula morphologies (33). OTSC yields favorable outcomes for fistula defects ≤10 mm in diameter. Its therapeutic effect is superior when used early after tissue injury, since fresh injury allows tighter closure. In contrast, chronically inflamed and fibrotic tissue tends to impede conventional repair procedures (31–33). The fistula diameter of 2 mm in this case fell perfectly within the optimal indication range for OTSC closure. The combination of APC-mediated de-epithelialization followed by OTSC deployment was fully consistent with these therapeutic principles. APC transformed this long-standing chronic fistula into fresh reparative tissue with exposed submucosal collagen. Subsequently, full-thickness tissue compression by OTSC facilitated immediate mechanical closure, permitting further granulation tissue proliferation and fibrous tissue healing, as observed during the 10-month follow-up.

This report has several inherent limitations owing to its single-case observational nature and relatively short 10-month follow-up period. Although the fistula remained closed at the latest evaluation, longer follow-up is necessary to definitively confirm permanent closure, rule out late recurrence, and evaluate the long-term impact on pulmonary function and nutritional status. Long-term prospective studies are required to fully validate the durability of endoscopic OTSC closure for congenital ADF, and to establish standardized patient selection criteria, including fistula size thresholds, anatomical indications, and the optimal choice of de-epithelialization techniques. Nevertheless, this case contributes valuable clinical evidence to support the minimally invasive treatment strategy for rare congenital ADF.

## Conclusion

This case highlights several important clinical insights for the management of congenital ADF. Firstly, congenital ADF may manifest unexpectedly in adulthood after decades of latent progression. Clinicians should maintain a high degree of suspicion in patients presenting with persistent, swallowing-induced cough, even without obvious congenital malformations. Secondly, multidisciplinary collaboration involving pulmonology, thoracic surgery, gastroenterology, radiology, and anesthesiology is essential for thorough evaluation and individualized treatment strategies. Thirdly, for eligible patients who are poor surgical candidates or decline surgical repair, combined endoscopic de-epithelialization and OTSC closure may represent a feasible minimally invasive treatment option in carefully selected patients with congenital BEF. Further prospective studies with larger patient cohorts and extended follow-up are warranted to validate the durability of this approach and to establish standardized patient selection criteria.

## Data Availability

The original contributions presented in the study are included in the article/Supplementary material, further inquiries can be directed to the corresponding author.
