# Supplementary material for: Congenital aerodigestive fistula with extraordinarily delayed adult presentation: a case report and review of endoscopic closure for bronchoesophageal fistula
Source: Front Med (Lausanne). 2026 Jul 2;13:1887528. doi: 10.3389/fmed.2026.1887528 (PMC13373083; doi:10.3389/fmed.2026.1887528)
Supplement: Supplementary file 1 [file Table_1.DOCX]

**Supplementary Table 1. Clinical Timeline:** **Endoscopic Closure of Congenital Bronchoesophageal Fistula**

| **Time Point** | **Event** | **Key Details** |
| --- | --- | --- |
| **~1984 (Age ~18)** | Symptom onset | Persistent swallowing-induced cough after liquid intake; white mucoid sputum production |
| **1984–2023 (40 years)** | Chronic course | Occasional symptomatic treatment at local clinics; no formal diagnostic workup; dietary restriction due to fear of coughing |
| **3 months pre-admission** | Symptom worsening | Increased frequency and severity of cough; sought definitive diagnosis |
| **Outside hospital (3 months pre)** | Initial CT | Left lower lobe mass suspicious for lung cancer with obstructive pneumonia |
| **Outside hospital** | Bronchoscopy | Esophageal–left main bronchial fistula identified; EBUS-TBLB: acute/chronic inflammation with fibrous hyperplasia |
| **Outside hospital** | Treatment | Moxifloxacin; patient refused nasogastric tube |
| **Day 0 (Admission)** | Transfer to institution | Repeat CT with MPR: direct upward fistulous tract (mid-esophagus to left main bronchus); Braimbridge & Keith Type II congenital BEF |
| **Day 3** | Diagnostic endoscopy | Bronchoscopy; 2 mm fistula orifice 2 cm below left main carina; methylene blue confirmation |
| **Day 4** | Multidisciplinary evaluation | Pulmonology, thoracic surgery, gastroenterology, radiology, anesthesiology consensus |
| **Day 5** | Therapeutic endoscopy | Combined bronchoscopy + esophagoscopy; APC de-epithelialization (40 W, 5-mm radius) + OTSC deployment (10 mm diameter, 6 mm cap, blunt tooth, transesophageal) |
| **Day 5–7** | Post-procedure | Immediate cough resolution; tolerated oral intake without aspiration |
| **10 months** | Follow-up | Esophagography: no contrast extravasation; CT: no persistent communication; Esophagoscopy: granulation tissue + white scarring, no fistula orifice |

Abbreviation: EBUS-TBLB: endobronchial ultrasound-guided transbronchial lung biopsy; MPR: multiplanar reformation; APC: argon plasma coagulation; OTSC: Over-the-Scope Clip; CT: computed tomography; BEF: bronchoesophageal fistula.
